# *In situ* formation of magnetopolymersomes via electroporation for MRI

**DOI:** 10.1038/srep14311

**Published:** 2015-09-22

**Authors:** Jennifer Bain, Lorena Ruiz-Pérez, Aneurin J. Kennerley, Stephen P. Muench, Rebecca Thompson, Giuseppe Battaglia, Sarah S. Staniland

**Affiliations:** 1 Department of Chemistry, University of Sheffield, Brook Hill, Sheffield, S3 7HF; 2 Department of Chemistry, University College London, Gordon Street, London, WC1H 0AJ; 3 MRC/UCL Centre for Medical and Molecular Virology, University College London, Gower Street, London WC1E 6BT; 4 Department of Biomedical science, University of Sheffield, Western Bank, Sheffield, S10 2TN; 5 SPiNSN, Department of Psychology, University of Sheffield, Western Bank, Sheffield, S10 2TN; 6 School of Biomedical Science, University of Leeds, LS2 9JT.

## Abstract

As the development of diagnostic/therapeutic (and combined: theranostic) nanomedicine grows, smart drug-delivery vehicles become ever more critical. Currently therapies consist of drugs tethered to, or encapsulated within nanoparticles or vesicles. There is growing interest in functionalising them with magnetic nanoparticles (MNPs) to target the therapeutics by localising them using magnetic fields. An alternating magnetic field induces remote heating of the particles (hyperthermia) triggering drug release or cell death. Furthermore, MNPs are diagnostic MRI contrast agents. There is considerable interest in MNP embedded vehicles for nanomedicine, but their development is hindered by difficulties producing consistently monodisperse MNPs and their reliable loading into vesicles. Furthermore, it is highly advantageous to "trigger" MNP production and to tune the MNP's size and magnetic response. Here we present the first example of a tuneable, switchable magnetic delivery vehicle for nanomedical application. These are comprised of robust, tailored polymer vesicles (polymersomes) embedded with superparamagnetic magnetite MNPs (magnetopolymersomes) which show good MRI contrast (R2* = 148.8 s^−1^) and have a vacant core for loading of therapeutics. Critically, the magnetopolymersomes are produced by a pioneering nanoreactor method whereby electroporation triggers the *in situ* formation of MNPs within the vesicle membrane, offering a switchable, tuneable magnetic responsive theranostic delivery vehicle.

Natural lipids (e.g. phospholipids) self-assemble to form biological membranes and vesicles (liposomes) and as such are traditionally employed in the formation of biocompatible vesicles for medical applications^1^. However phospholipids can prove restrictive and unstable with respect to responsive functionalization limiting their use in new smart nanomedical materials. More recently, amphiphilic copolymers have been utilized as lipid mimics. Their self-assembly into “polymersomes” (polymer vesicles) under a range of chemical conditions offers a more robust and flexible system with greater scope for modifications both pre and post formation. This, in combination with the intrinsic improvement of the final robust mechanical properties, makes polymersomes ideal drug delivery and diagnostic agents^2^,^3^.

The applicability of a therapeutic or diagnostic agent can be dramatically enhanced by the addition of magnetic functionality, achieved by incorporation of magnetic nanoparticles (MNPs). Post formation loading of MNPs into a range of vesicles and particles is of rapid growing interest for use in a range of targeted medical treatments^4^,^5^,^6^,^7^,^8^,^9^. MNP incorporation into vesicles can be localized and concentrated to a specific site using a large magnetic field^10^, making magnetic vesicles ideal targeted drug-delivery vehicles^11^,^12^,^13^,^14^. They can be engineered to release cargo at a specific treatment site, allowing for lower therapeutic doses which significantly improves treatment efficiency, whilst simultaneously reducing overall dose and side effects^15^. Magnetized vesicles are also valuable contrast agents in existing diagnostic techniques such as MRI imaging, as their local magnetic field dramatically increases the local atomic relaxation time, resulting in darkening of this area of the image. Furthermore, the magnetism can be exploited for therapeutic magnetic hyperthermia treatments, in which MNPs exposed to a time varying magnetic field gives rise to magnetically induced heating, and the subsequent destruction of surrounding tissue and/or release of heat activated drugs, which have the potential for both conjugation to the polymersome exterior or to be encapsulated within the vesicle core^10^,^16^,^17^. All these elements can also be combined to give a multipurpose “theranostic” nanomedicine. The amount of heat generated (hyperthermia), and the degree of contrast achieved (MRI) is highly dependent on MNP characteristics such as the size and concentration of the particles^10^,^18^.

It is clear that the combination of a robust, modifiable polymersome with the magnetic response of MNPs could form an ideal theranostic delivery agent for nanomedicine. Encapsulated MNPs within polymer supported liposomes and polymersomes have recently been investigated for both drug delivery^19^ and for theranostic nanomedicine^9^, respectively. However, loading of MNPs into the vesicle core along with therapeutics, results in adverse effects such as simultaneous release of MNPs and drugs. Furthermore, the magnetopolymersomes developed to date rely on a multistep process of crystallising and processing MNPs, then subsequent loading into polymer vesicles^9^,^19^. For this crystallisation step, iron-oxide MNPs can be produced by various methods leading to differing size, shape and population heterogeneity^18^. The simplest route to magnetite MNPs is a room temperature co-precipitation, whereby MNPs are precipitated by raising the pH of a mixed valence iron solution with base (e.g. NaOH) under an inert atmosphere. However, in the absence of any additives or templates, the resulting MNPs are of a broad range of shapes and sizes and commonly contain a range of other iron-oxide contaminants. Mixed populations require labour-intensive sorting before they are suitable for loading into the vesicles^9^. Other methods produce more mono-disperse populations, but require additional additives, toxic and/or organic (non-biocompatible) solvents /reagents and high temperatures, resulting in costly, multistep processes^18^. Consequently, current magnetopolymersomes are not ideal with respect to easy synthesis and the challenge remains to create a magnetopolymersome with: mono-disperse MNPs embedded in the membrane, leaving the core free for therapeutic agents to be released; via a simple method involving mild conditions that offers precise control over MNP size and concentration. We overcome the issues outlined above and achieve this goal by using electroporation to initiate *in situ* production of magnetopolymersomes with a mono-disperse population of MNPs within its polymersome membrane.

More generally, electroporation uses an electric field to open temporary hydrophilic nanoscopic pores in a cell or vesicle membrane allowing reagents to cross the membrane^20^,^21^,^22^,^23^. The potential generated across the membrane is dependent on the membrane composition, cell size, and extracellular medium^20^. Until recently electroporation has only been used to open pores in lipid membranes to allow the insertion of biomolecules into biological cells, (e.g. insertion of DNA into host cells)^21^.

Recently we reported the first use of electroporation to open pores in polymer membranes to load polymersomes with nucleic acids and proteins post vesicle formation, proving that polymer membranes cannot only withstand electrical stress but are also able to undergo electroporation, and remain functional^22^.

Here we report a completely new synthetic method of magnetising vesicles using electroporation, marking a novel and previously unreported use of this technique. Polymersomes with a high pH aqueous core are suspended in an iron solution with the membrane providing a reaction barrier. We propose a mechanism whereby upon electroporation, pores open allowing synthetic ion-transport across the polymer membrane, resulting in an iron ion/base interface within the poration channels. The membrane acts as a nucleation site and the pore acts as a template to restrict the size of the growing MNPs within the membrane (Fig. 1). We have, for the first time applied electroporation to initiate a nanoreactor chemical precipitation of inorganic reagents; offering greater templated control in an easy, one-step process. Control of the electroporation parameters can control pore size and thus iron flow, regulating MNP size, resulting in the advantage of magnetically tuneable magnetopolymersomes. Furthermore, the *in situ* electroporation process offers a switchable magnetic response activated by an electric field. In this study, polymersomes encapsulating OH^−^ ions are electroporated in the presence of mixed valence iron salts. Electroporation allows the permeation of reagents, resulting in *in situ* crystallization of MNPs within the polymersome membrane yielding magnetopolymersomes. These can then be purified ready for further application. This opens up extensive engineering opportunities with respect to MNP/polymersome properties for various applications.

Our new approach to the synthesis of magnetopolymersomes permits the creation of a simple biocompatible nanoreactor, activated by the application of an electric field for the engineering of precise magnetopolymersomes.

## Results and Discussion

The amphiphilic block copolymer was synthesized by the polymerization of ethylene oxide (PEO) and butadiene (PBD) monomers. PBD (1300)-PEO (2500) was chosen for polymersome synthesis as previous studies had shown the production of monodisperse unilamellar vesicles, within the required size range for this system^24^,^25^,^26^. Polybutadiene is a robust non-polar monomer, making it ideal for the electroporation process, as it can withstand the application of an electric field without degradation. In contrast the polyethylene oxide is polar, yet biocompatible and widely used to extend the physiological lifetime of several biomedical nanoparticles (NPs) and vesicles. Polymersomes were produced simply by rehydration and swelling of a polymer film (Methods). PBD-PEO forms vesicles with a bilayer of 4–5 nm (Supplementary Figure S1) comparable to a naturally occurring lipid bilayer. Additionally, extrusion was used to explore vesicle size control. Transmission electron microscopy (TEM) and Dynamic light scattering (DLS) analysis were used to characterize both the polymersome size and the sample size distribution (Fig. 2). These techniques are complementary: Transmission Electron Microscopy (TEM) of stained non cryo preserved samples shows the morphology, degree of aggregation, and sample homogeneity, but staining and drying artefacts can result in distortions and an appearance of shrinkage of the vesicles. DLS offers universal analysis of the complete sample (accurate population), but as a scattering technique gives the full hydrodynamic size, and uses a parameter defined for a perfectly spherical material, which is a not an exact match to our polymer or vesicle geometry, both of which contribute to them appearing larger than the true polymersome size. Therefore, it should be noted that there is a discrepancy (approximately 2-fold) between the DLS and TEM sizing, but importantly the population distribution is in agreement. With this in mind, cryo-EM was also performed (Fig. 3) in which the images, which do not suffer the same deformations from staining and drying, show a preserved membrane bilayer and a true size slightly larger than the stained TEM; however, cryo-EM cannot be performed on the large scale required to obtain a size distribution. Importantly, the TEM analysis of the stained sample permitted a rapid and clear assessment that the samples are of high homogeneity and low aggregation. It was found that while extrusion produced a close to homogeneous population of 41.2 ± 6.4 nm by TEM (123 ± 26 nm by DLS) it does require an extra processing step, which may not be necessary as a simple stirred method yields two populations of polymersomes of a similar polydispersity of 100.9 ± 8.6 nm by TEM (203 ± 55 nm by DLS) (Fig. 2A), with an additional population around 43 nm by TEM (58 nm by DLS). The polydispersity of this stirred sample improves as a matter of course in later processing of the magnetopolymersomes (size separation via clean up on the column, Supplementary Figure S2). The similar distribution achieved by a simple method such as stirring alone is indicative of the simplicity of this process, further highlighting its advantage over other synthetic routes.

We successfully encapsulated a range of different bases of varying ionic strength and 10 mM NaOH encapsulated polymersomes (Fig. 2) were found to be the lowest optimal base concentration for an instantaneous room-temperature precipitation of MNPs. Below this concentration range heterogeneous, mixed crystalline and amorphous iron oxides are precipitated (Supplementary Figure S3). Low ambient concentrations of reagents are favoured in keeping with greener routes, as well as ensuring little to no effect on the polymersome formation or toxicity in later *in vivo* use. Moreover, this simple synthetic route occurs under ambient conditions, whereas other bases tested (KOH and CO(NH_2_)_2_) required sustained heating for MNP formation making them impractical for vesicular MNP precipitation. 10 mM base offered the optimal balance between good MNP precipitation and polymersome stability.

When base filled polymersomes were suspended in an iron solution, MNPs did not form, showing that the natural permeability of the PBD-PEO membrane was not sufficient to allow the reagents to penetrate the membrane (i.e. the base cannot leak out and the iron ions cannot leak in) (Fig. 3A). However when these polymersomes were subjected to electroporation (2500 V, 5 Pulses) reagents were able to diffuse through the resulting membrane pores and MNPs were formed within the membrane successfully. The MNP size analysis showed them to be 2.54 nm in diameter with a tight size distribution (SD ± 0.46 nm) (Supplementary Figure S4) at this voltage. Energy dispersive x-ray (EDX) elemental analysis showed the particles were iron-oxide species by confirming the presence of iron and oxygen (Supplementary Figure S5). Induced coupled plasma emission spectroscopy (ICP-ES) elemental analysis reveals iron compounds made up approximately 1.40–1.42% (dependent on mineral type) of the magnetopolymersome (iron: total polymer ratio (Supplementary calculation 1)). Magnetic hysteresis measurements reveal the particles to be superparamagnetic iron oxide and that MNP magnetic material makes up approximately 0.20–0.23% of the magnetopolymersome mass which is comparable but suggests that the iron present may not all be magnetic mineral. In order to determine how the MNPs are associated with the membrane cryo-EM analysis was employed (Fig. 3) to ensure no preparation artefacts^27^. Cryo-EM images of unelectroporated polymersomes (Fig. 3A) and electroporated magnetopolymersomes (Fig. 3B) showed particles present within and associated with the membrane only in cases where electroporation had been applied with negligible free particles in the background (Fig. 3). Size analysis of the magnetopolymersomes based on the room temperature TEM data showed them to be 47.26 ± 9.29 nm (Fig. 3C, Supplementary Figure S2). To further characterize the vesicles and their associated MNPs a tomographic tilt series of the polymersome sample was generated allowing for a clearer analysis of both the polymersome membrane and its interior, again showing no MNPs in the core of the vesicle (Supplementary Video). The cryo EM images (Fig. 3A,B) were obtained 10 months after the sample was prepared as opposed to the TEM image in Fig. 3C which was obtained 2 months after preparation, which can explain some of the differences seen between the images. After a period of time, any MNPs released as a result of degradation will be attracted to the exterior of other magnetopolymersomes which is seen in Fig. 3B. It is noteworthy that the level of degradation is very low showing good stability of the magnetopolymersomes over a long time period.

Further to this, a pH analysis was conducted at each stage of the process and showed minimal change in pH to the extra-vesicular solution before and after electroporation (Supplementary Figure S6) showing all the NaOH is reacted and neutralised during the MNP formation process. Polymersomes were also sonicated to disrupt the membranes before and after electroporation. Disruption of base filled polymersomes that had not undergone MNP precipitation showed an increase in pH as the NaOH was released. This is contrary to the disruption of the magnetopolymersomes, which showed a small lowering of the pH. This is further evidence that no NaOH remains inside the interior after electroporation and the small difference shows that the insertion of MNPs within the membrane does not alter the integrity of the membranes. Importantly, the small pH changes seen are not significant enough to raise toxicity concerns for eventual *in vivo* applications.

**Figure 1:**
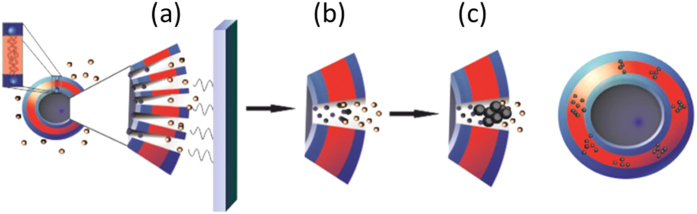
Schematic of the proposed mechanism for MNP synthesis within polymersomes using electroporation. (**a**), which opens pores within the membrane at which point influx of iron ions occurs in parallel with efflux of NaOH (encapsulated) (**b**). (**c**) shows the *in situ* room temperature co-precipitation that then occurs at the interface within the membrane.

**Figure 2:**
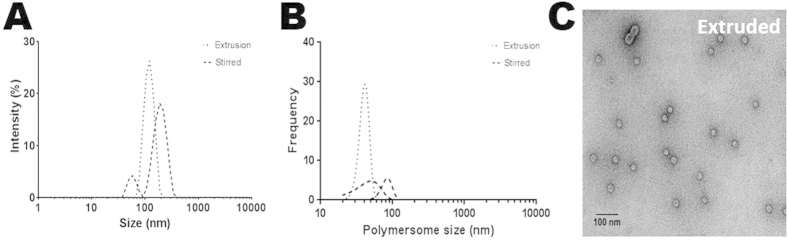
Characterisation of the polymersomes. Comparison of two different preparation methods of the NaOH encapsulated polymersomes; Extruded and stirred. (**A**) DLS data sizing (**B**) the corresponding TEM grainsizing and (**C**) TEM image of an extruded polymersome sample (stained with 0.75% uranyl formate) (cryo-TEM image of stirred sample shown in Fig. 3A).

**Figure 3:**
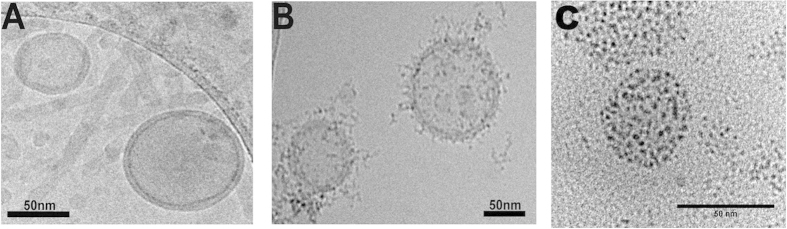
Analysis of magnetopolymersomes. Single particle cryo-EM images of extruded polymersomes before (**A**) and after (**B**) electroporation, with the latter displaying a high density of iron particles around the membrane. (**C**) shows the same sample as in (**B**) as imaged by room temperature TEM.

A small number of polymersomes can be seen to have smaller polymersomes encapsulated within them. From the analysis of these samples it is clear that precipitation of MNPs is limited to within the outer membrane, evident by the lack of MNPs within the membrane of the inner polymersome (Supplementary Video). This provides further evidence for our hypothesis for the mechanism of precipitation presented in Fig. 1. It is also highly advantageous as it leaves the core of the vesicle completely free for further functionalization, such as the encapsulation of therapeutics during formation or post formation attachment to the outer PEG monomer. This study has shown that polymersomes can be functionalized *in situ* by a mechanism which can be universally applied to inorganic NP mineralization within a nanoreactor.

**Figure 4:**
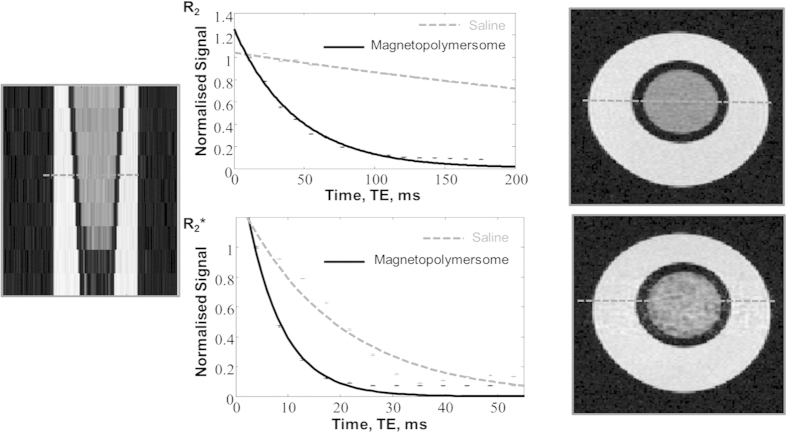
Magnetopolymersomes show enhanced contrast in MRI imaging. 7 T magnetic resonance R2 relaxometry data (16 echoes, 9 slices, image shows 5th slice) and 7 T magnetic resonance R2* relaxometry data (12 echoes, 9 slices, image shows 5th slice). Both R2 and R2* relaxometry rates show significant differences between saline (dashes) and electroporated polymersomes (solid).

Magnetic resonance (MR) relaxometry results (at 7 Tesla) for the R_2_ and R_2_* rates of our magnetopolymersomes in solution were 22.7 s^−1^ and 148.8 s^−1^ respectively (Fig. 4). The R_2_ relaxivity normalized for iron concentration was 9.1 [Fe mM]^−1^ s^−1^. Detailed analysis conducted on both magnetopolymersomes and unelectroporated polymersomes show a distinct difference in R_2_ relaxometry, with the addition of MNPs in the polymer membrane showing a significantly faster decay with increasing magnetopolymersome concentration (Supplementary Figure S7). The decay of the control unelectroporated polymers is comparable to that of the saline control sample confirming that little or none of the contrast shown in Fig. 4 is the result of signal decay as the protons diffuse in and around the polymersome membrane. Similarly, R_2_* control data (Supplementary Figure S7) shows enhanced decay with the presence of MNPs, although it was not possible to extract a clear relationship between MNP concentration and decay due to the very rapid relaxation. It is clear that the precipitation of small superparamagnetic particles within the membrane has positive effects; firstly as the precipitation of 2.54 nm particles has a much greater surface area than more conventional MNPs of about 20 nm; and secondly on the unavoidable, but often useful diffusion effects we see in the R_2_ decay data. The position of MNPs within the membrane immobilises them in close proximity to the water molecules, essentially creating more random magnetization dephasing interactions that cannot be refocused with conventional spin-echo (MR) techniques. The formation method presented here also has the potential to be tuned to give magnetopolymersomes with differing numbers and sizes of MNPs by varying a number of parameters (Supplementary figure S8). For example, varying the electroporation voltage between 250 V and 2500 V changes the size and number of MNPs per polymersome (Supplementary Figure S8a) with larger MNPs forming at lower voltages, and an increased R_2_ relaxivity per mM Fe concentration is seen from 1.3 [Fe mM]^−1^s^−1^ to 9.1 [Fe mM]^−1^s^−1^ (Supplementary Figure S8b). We also observe a strange crossover in parameters at 1000 V where we see smaller particles in increased number (Figure S8). We are currently trying to understand the mechanism by which this occurs and optimize the parameters to tune for the full range of specific MNP sizes and MRI signal.

Peters *et al.*^28^ measured the grey matter R_2_* relaxation rate as 30 s^−1^. This is significantly different to the R_2_* relaxation rate for our vesicles (148.8 s^−1^) suggesting they could be very promising future *in vivo* biomedical agents; however this will require further dose and toxicity related investigation in a pre-clinical setting.

## Conclusion

A method has been developed for the *in situ* formation of superparamagnetic iron oxide NPs within a polymersome. The data confirmed that MNP precipitation occurred in the vesicle membrane. We propose a mechanism (Fig. 1) whereby pores formed by electroporation enable the simultaneous efflux of base and influx of iron through the pores to mix the reagents and equilibrate the pH gradient within the membrane, yielding the crystallization of multiple membrane embedded MNPs templated within the pore. The diameter of magnetopolymersomes was shown to be homogeneous by DLS and TEM with no signs of aggregation with embedded magnetite MNPs of 2.54 nm in size (Supplementary Figure S4). Further optimization of the reactant concentrations should alter the rate of influx and efflux of substances across the membrane, due to the basic principles of osmosis and diffusion; this in turn will have the potential for further regulation of MNP formation.

The advantages of this method are many: Firstly the simplicity of the whole system. The polymersomes are easy to produce in stable narrow-size distribution populations, and the MNPs are produced simply in a one-step *in situ* process. Secondly, the precipitation reaction is a room temperature route that offers vast scope for tuning and optimising the material. The method offers flexibility with respect to tuning the number and sizes of the MNPs within the membrane simply by altering the reagent concentration and/or the electroporation parameters. Thirdly, the non-specific nature of electroporation offers immense flexibility for this method to be developed and optimized for the incorporation of a wide range of inorganic NPs (magnetic or otherwise), from simply doping the MNPs (to tune the magnetic properties) to precipitating alternative MNPs such as CoPt or NPs with different properties such as quantum dot fluorescent markers. Fourthly, the use of electroporation allows for the remote triggering of MNP precipitation by application of an electric field. This is hugely advantageous, mainly from a practical point of view in that MNPs can be highly sensitive to oxidation and can incur stability problems. Electroporation at the point of use delivers the freshest MNPs to ensure the timely delivery of high quality, stable MNPs for application, removing shelf-life issues. Additionally, remotely triggering polymersomes to form magnetopolymersomes via electroporation can present a host of opportunities in further applications, for example, in cases were a magnetic response would be valuable during application both *in vivo* and *in vitro* such as in the case of iron sensing assays. Fifthly, the structure of the magnetopolymersome in which MNPs are located bound within the membrane leaves the vesicle core free for encapsulation during formation and subsequent localised release of hydrophilic drugs, showing their great potential as therapeutic agents. Finally, our magnetopolymersomes have proved to provide good contrast during MRI imaging trials which shows they could prove to be viable for *in vivo* diagnostic application (following further optimization and toxicity studies).

We present a significant first step towards realizing a fully tuneable and remotely switchable theranostic agent, in which MNP formation within a membrane can be controlled *in situ* by application of an electric field. Although the methods presented in this work are specific to iron oxide formation, electroporation controlled NP synthesis could open up new methods for a wide range of NP embedded polymersome syntheses.

## Methods

### Polymersome formation

Polymer was purchased from Polymer Source Inc. (Canada) and can be synthesised using living anionic polymerisation according to the method previously published by Hillmyer *et al.*^24^ Polymer species were confirmed by nuclear magnetic resonance (NMR) (Supplementary Figure S9). All other reagents were purchased from Sigma Aldrich (UK). Unilamellar vesicles were prepared by dissolution of amphiphilic block copolymer polyethylene oxide-polybutadiene (PBD (1300)-PEO (2500), 1–4 addition, 10 mg) into chloroform (1 ml). Subsequent evaporation under vacuum at room temperature of the solvent resulted in a polymer film across the bottom of the vial which was rehydrated with a variety of 10 mM basic solutions to encapsulate a basic core. Three methods were then used to form polymersomes. 1. The rehydrated film solution was stirred over 24 hrs. 2. The rehydrated film solution was tip sonicated at room temperature for 30 minutes on 50% amplitude. 3. The rehydrated film solution was extruded though a filter containing 100 nm pores 15 times.

### *In situ* electroporation of polymersomes

Due to the sensitivity of the iron salt to oxidation, all solutions were degassed prior to use and experiments were carried out under an inert atmosphere. Polymersomes encapsulating NaOH solution (10 mM) were passed through a Sephadex column containing Sepharose 4B to remove the basic supernatant to avoid the formation of external precipitation on addition of iron solution. Polymersomes at a concentration of approximately 1 mg/ml (as measured by gas chromatography (Supplementary Figure S10)) containing NaOH and suspended in PBS buffer were added (in a 1 ml 1:1 volume ratio) to an excess volume of mixed valence iron salt solution (containing FeCl_2_.4H_2_O (10 mM) and FeCl_3_.6H_2_O (10 mM) in a 1:2 (v/v) ratio). The solution was electroporated (Eppendorf (Eporator), Bio-rad (Multipulser), 5 x at 2500 V pulse length averaging 2.4 ms) in an excess of mixed valence iron salt solution to open up pores within the membrane. Following electroporation the polymersomes were passed back through a Sephadex column to remove excess iron solution and external iron oxide precipitates which were not tightly associated with the polymersome.

### Characterisation of Magnetopolymersomes

Polymersome size was initially evaluated using dynamic light scattering (Malvern Zetasizer (Zetasizer Nano)). Formation, evidence of encapsulation and size analysis was evaluated using transmission electron microscopy (Philips CM200,) as well as energy dispersive X-ray analysis for elemental characterisation (Supplementary Figure S5). Cryogenic transmission electron microscopy (cryo-EM) was used to directly visualise the resulting polymersomes. Grids were prepared by placing 3 μl of sample onto a glow discharged 300 mesh Lacey carbon grid. The grids were subsequently blotted for 6 seconds (at 100% humidity) and plunged into liquid ethane using a Vitrobot mark IV. Samples were visualised on a Tecnai F20 microscope fitted with a Gatan 4 k × 4 k CCD camera. All data was collected in low dose mode. Single axis tilt series were collected using SerialEM^29^ with an object sampling of 0.44 nm per pixel. Tilt series were collected (from +/−30^o^ for the supplementary movie) at 2^o^ increments using a dose of ~2 e^−^/Å^2^. Tilt series were collected at ~6 μm defocus. Tilt series were reconstructed by fiducial-less alignment in 3DMOD, part of the IMOD package^30^. Elemental composition of magnetopolymersomes was confirmed with inductively coupled plasma emission spectrometry (ICP-ES) by dissolving a magnetopolymersome samples in 1 M nitric acid to remove the organic polymer material from around the nanoparticles. This was then related to the magnetic saturation of the magnetopolymersomes as measured with room temperature superconducting quantum interference (SQUID) magnetometry, on a film of magnetopolymersomes at 1 T (300 K).

### *In vitro* MRI measurements

The inclusion of superparamagnetic nanoparticles within the polymersome membrane makes them ideal candidates for future magnetic resonance (MR) contrast agents or traceable *in vivo* therapeutic transporters. To test the applicability of our magnetopolymersomes, MR relaxometry was undertaken to quantify the transverse relaxation rates (R_2_ & R_2_*). An Eppendorf containing 1 ml of magnetopolymersomes (of initial concentration of 10 mg/ml polymer before synthesis and purification) was placed inside a tube of saline (for improved field shimming purposes) and this was placed at the iso-centre of a 7 Tesla magnet (Bruker BioSpec Avance III, 310 mm bore, MRI system B/C 70/30). The system used a 660 mT/m imaging gradient set with integrated shimming coils (BGA-12S Bruker, Germany). A ^1^H quadrature resonator (300 MHz, 1 kW max, outer diameter 114 mm/inner diameter 86 mm) was used for both transmission and reception of RF. Transverse relaxation rates, R_2_ & R_2_*, were measured using a Multi-Spin-Echo and Multi-Gradient-Echo sequence, respectively. Image matrix size was 256*256, FOV = 40 mm, slice thickness = 1 mm. Nine slices were taken through the images covering the full depth of the Eppendorf. For R_2_ estimation TR = 2.5 s, minimum TE = 11 ms increasing in steps of 11 ms for 16 echoes. For R_2_* estimation TR = 2.5 s, minimum TE = 3.8 ms increasing in steps of 4.8 ms for 12 echoes (excitation flip angle = 60 degrees). Post experiment image data were analysed in Matlab (Mathworks). ROIs were selected within the Eppendorf and the saline (control) and the signal as a function of echo time was fitted using non-linear-least squares to a simple mono-exponential decay function to estimate the relaxation rate.

## Additional Information

**How to cite this article**: Bain, J. *et al.* *In situ* formation of magnetopolymersomes via electroporation for MRI. *Sci. Rep.* **5**, 14311; doi: 10.1038/srep14311 (2015).

## Supplementary Material

Supplementary Information

Supplementary video
