# Supplementary material for: In situ formation of magnetopolymersomes via electroporation for MRI
Source: Sci Rep. 2015 Sep 22;5:14311. doi: 10.1038/srep14311 (PMC4585761; doi:10.1038/srep14311)
Supplement: Supplementary Information [file srep14311-s1.pdf]

Supplementary Information.

***In situ* formation of magnetopolymersomes via electroporation for MRI**

Jennifer Bain[†], Lorena Ruiz-Pérez[‡, ±,β], Aneurin J Kennerley[⊥], Stephen P. Muench[#], Rebecca Thompson[#], Giuseppe Battaglia[‡,±,β], Sarah S. Staniland[†]*

[†]Department of Chemistry, University of Sheffield, Brook Hill, Sheffield, S3 7HF. [‡]Department of Chemistry, University College London, Gordon Street, London, WC1H 0AJ. [±]MRC/UCL Centre for Medical and Molecular Virology, University College London, Gower Street, London WC1E 6BT. [β]Department of Biomedical science, University of Sheffield, Western Bank, Sheffield, S10 2TN. [⊥]SPiNSN, Department of Psychology, University of Sheffield, Western Bank, Sheffield, S10 2TN. [#]School of Biomedical Science, University of Leeds, LS2 9JT.

Corresponding author email address: s.s.staniland@sheffield.ac.uk

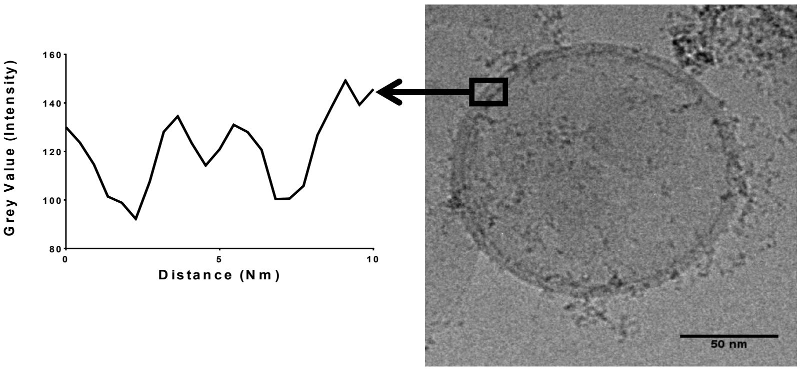

Supplementary Figure S1: Polymersome bilayer measurement. Greyscale measurement of polymersome bilayer showing a thickness of 4-5nm.

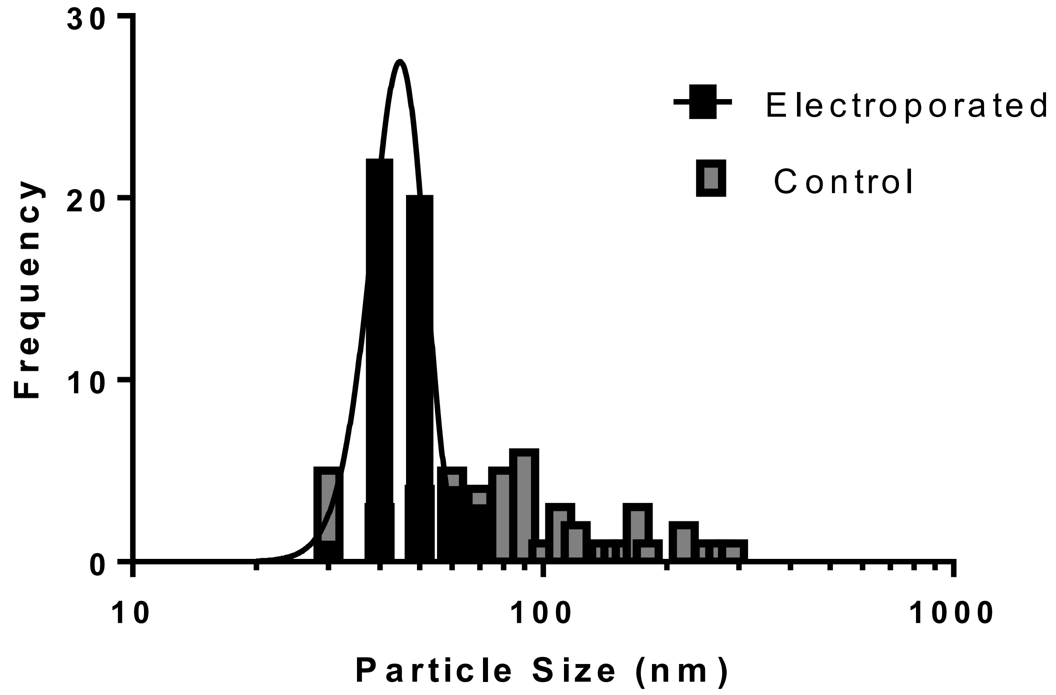

Supplementary Figure S 2: TEM sizing distributions of a control sample and a columned and electroporated sample showing a narrower size distribution in the electroporated sample as a result of size separation during column clean up.

$$Fe^{2+} + 2Fe^{3+} + 8OH^- \rightarrow 2\beta FeOOH + Fe^{2+} + 2H_2O + 2OH^- \rightarrow Fe_3O_4 + 4H_2O$$

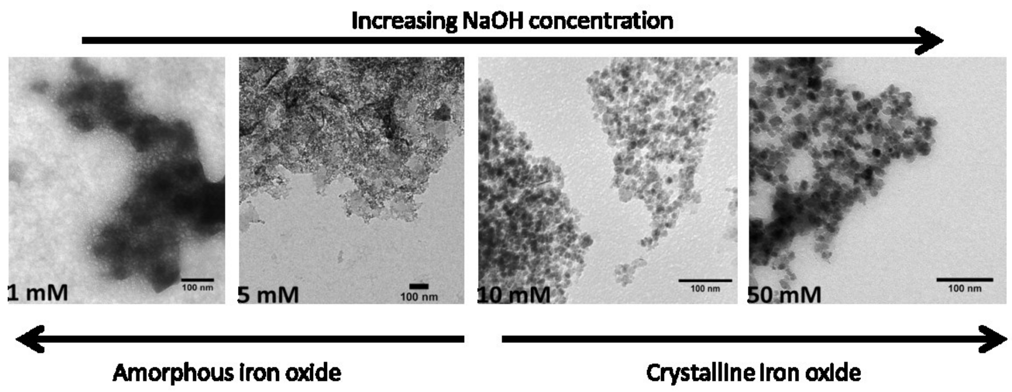

Supplementary Figure S3: TEM analysis MNPs produced with increasing NaOH. Room temperature co-precipitation (equation; top) reactions carried out at NaOH concentrations of 1 mM, 5mM, 10 mM and 50 mM. Below 10 mM we see only amorphous iron oxide at 10 mM and above the iron oxide becomes crystalline forming discrete particles.

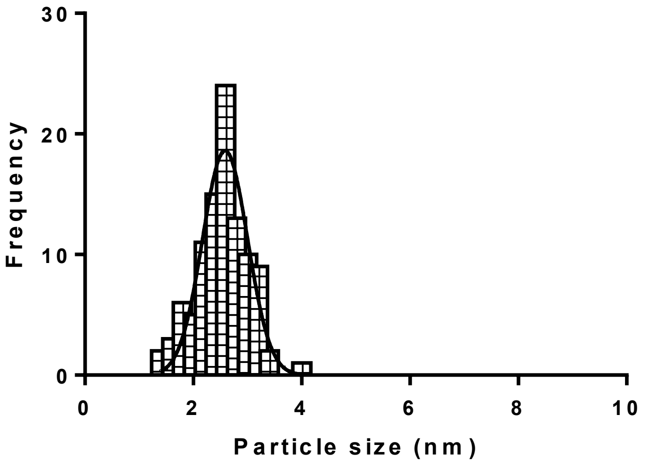

Supplementary Figure S4: Magnetic nanoparticle grainsizing. Taken from TEM images shows the particles to have an average diameter of 2.54 nm (SD ± 0.46 nm).

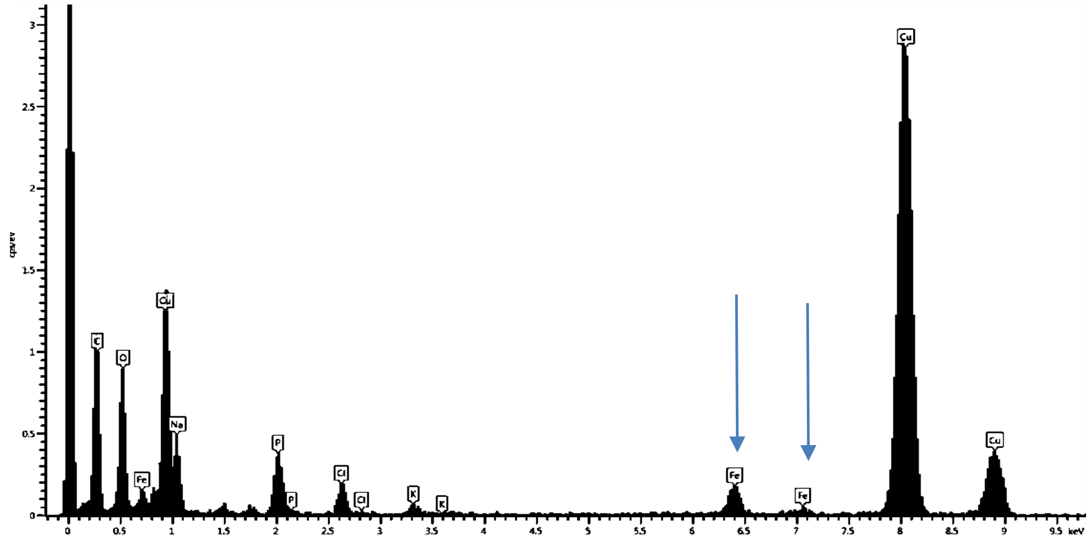

Supplementary Figure S5: Presence of iron oxides detected within magnetopolymersomes. Energy dispersive x-ray spectrum of an MNP within the bilayer of a magnetopolymersome. Spectrums show copper at 8 and 9 keV as a result of the copper mesh TEM grids. Presence of Iron oxide confirmed by peaks for both iron (6 – 7,5 keV) and O (0,5 keV).

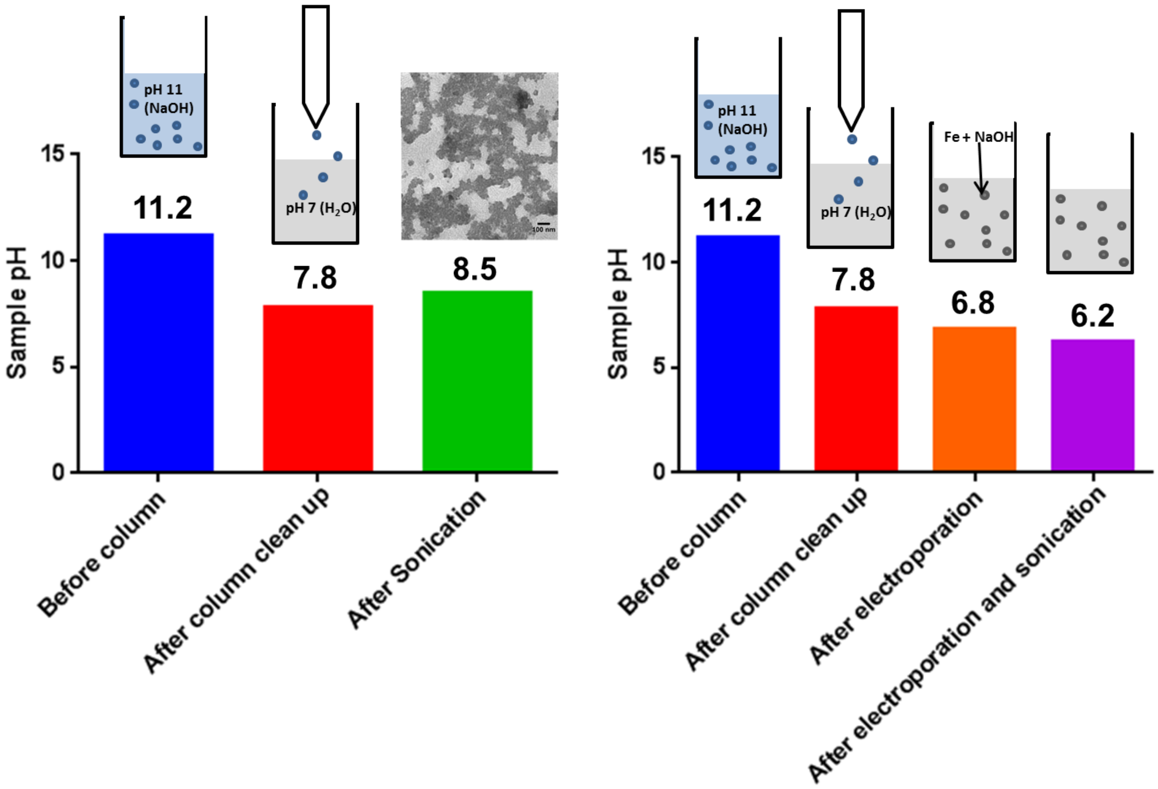

Supplementary Figure S6: Leakage of NaOH during polymersome preparation. The pH of a polymersome solution (10 mg/ml) was taken before (blue column) and after (red column) passing through a sephadex column, to ensure effective removal of external NaOH, the sample was then sonicated to break open the polymersome; as shown by the TEM. The pH was then taken again to determine the effect of release of the polymersome encapsulant on the overall solution pH (green column); little change was observed. A second electroporated sample was also tested first as with the control sample before and after clean up to ensure effective removal of external NaOH (blue and red columns as before). The polymersomes were then electroporated and again passed back through the column to removes external iron solution, at this point the solution pH was taken again (orange column). Finally the now magnetopolymersome sample was sonicated to break open the polymersome and release any cargo (to simulate potential drug delivery) as well as any residual/unreacted NaOH and the pH taken a final time to measure any change to the overall pH as the result of this action (purple column). We see minimal and a lower change in pH after the magnetopolymersomes have been cleaned up post electroporation, with a further minimal lowering of the pH post sonication indicating no NaOH remains post MNP production.

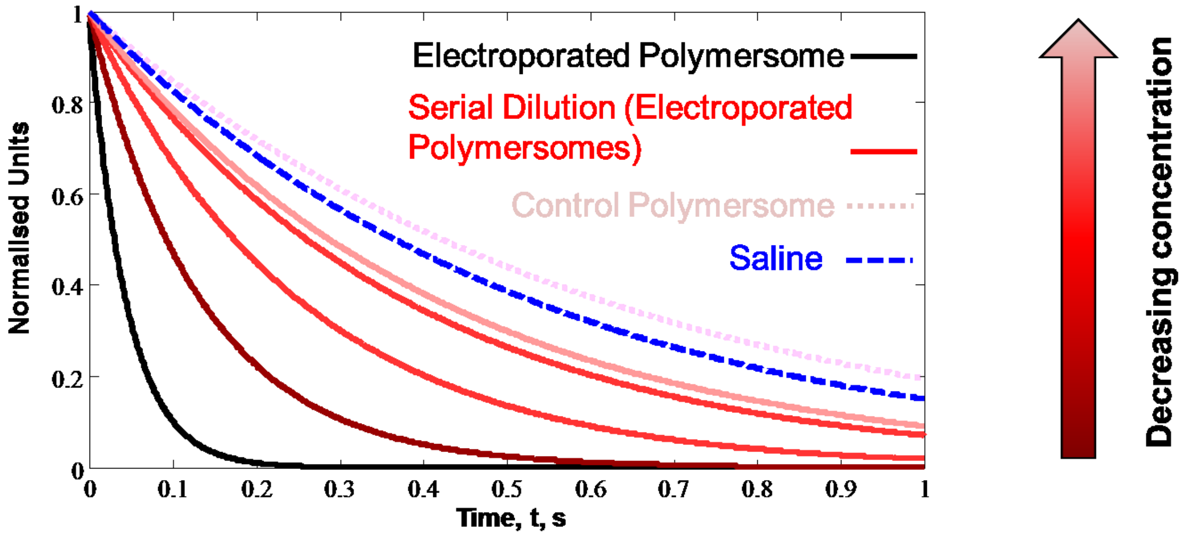

Supplementary Figure S7: Effect of mangetopolymersome on enhanced MRI contrast. Control tests conducted on magnetopolymersomes, unelectroporated polymersomes and saline samples show a difference in R_2 relaxometry upon addition of MNPs in the polymer membrane. Electroporated polymersomes display a significantly faster decay than saline and unelectroporated controls. The decay is dependent on magntopolymersome concentration, becoming slower as the concentration of magnetopolymersomes decreases. The decay of the control unelectroporated polymersomes is comparable to that of the saline sample confirming that little or none of the contrast shown in Figure 3 is the result of signal coming from the polymersome membrane.

R_2* control data shows faster decay with the presence of MNPs, although it was not possible to extract a clear relationship between MNP concentration and decay. This is due to the decay being faster than the time window needed to collect sufficient data points to form a suitable exponential decay, therefore the control R_2* can only offer us a qualitative conclusion at this point.

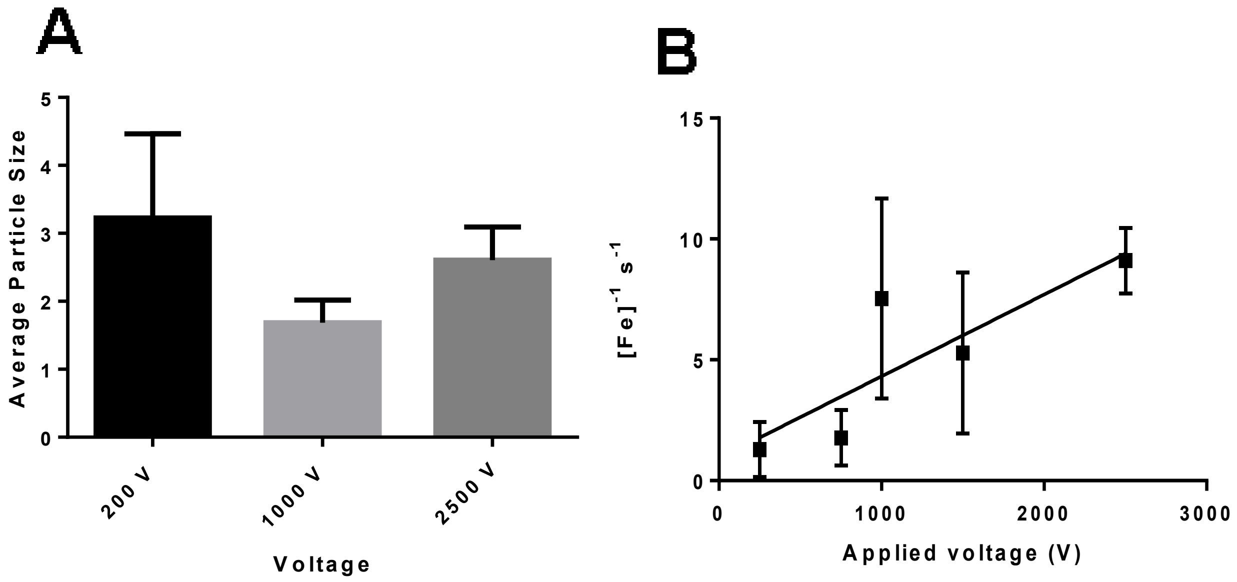

Supplementary Figure S8 Change in particle size with electroporation. A Plot shows the average particle size at 200, 1000, and 2500 V. **B** Plot shows that as we increase voltage applied during electroporation we increase the iron concentration and consequently the relaxivity.

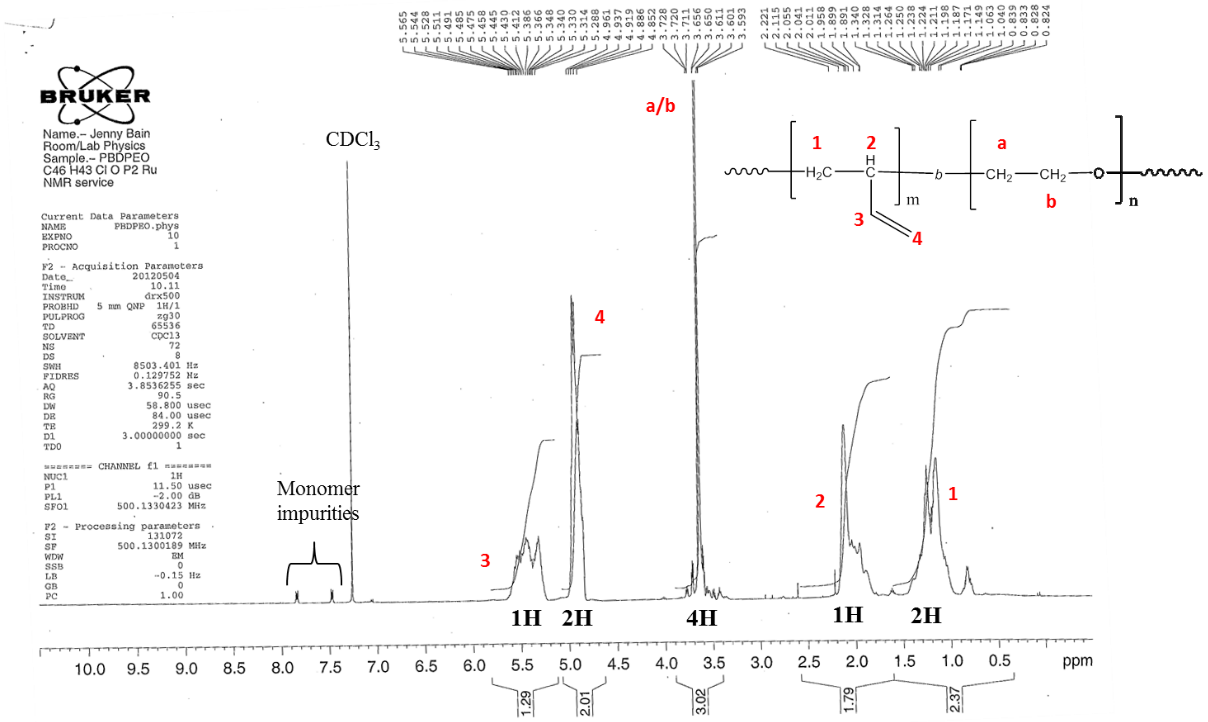

Supplementary Figure S9: NMR analysis of block co-polymer. A nuclear magnetic resonance spectrum of block copolymer PBD-PEO used for polyermersome formation was taken before the polymer was utilised in film rehydration synthesis. NMR spectrum confirms expected structure for PBD-PEO (1-4 addition).

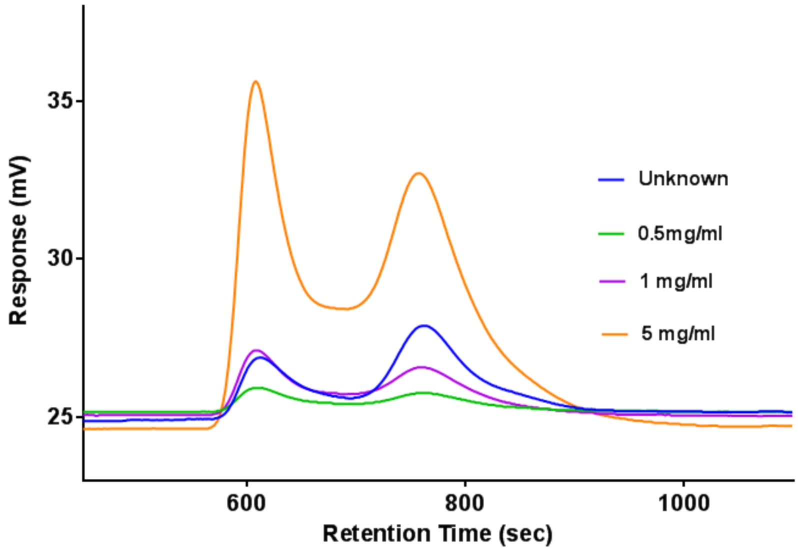

Supplementary Figure S10: Gas chromatography calibration for determination of polymersome concentration. Carried out after dialysis and column clean-up for removal of the basic supernatant prior to iron electroporation.

Supplementary Video: Tomographic analyses of MNP position with magetopolymersomes.
Tomography video attached

Supplementary calculation 1. Estimation of magnetic material:

Magnetopolymer film mass $= 0.7\ mg$

Gas chromotography polymer concentration $= 0.2\ mg/ml$

Polymersome area $= 4\pi\ x\ 2.5\ x\ 10^{-7} = 1.57\ x\ 10^{-65} cm^3$

Volume of Polymer (x) $= \frac{2x\ 10^{-4}}{1.11}$

N x Area $= x\ 1.8\ x\ 10^{-4}$
N° of vesicles $= \frac{x}{Area} = \frac{1.8\ x\ 10^{-4}}{1.57\ x\ 10^{-65}} = 1.146\ x\ 10^{61}$

Therefore volume occupied $= n\ x\ 100\ nm^3$

Magnetic film total mass $= 0.7\ mg/ml$
Polymer mass $= 0.2\ mg/ml$

Therefore theoretical internal volume $= 0.7 - 0.2 = 0.5\ mg/ml$
$4\pi r^2\ x\ 5 \ :\ \frac{3}{4}\pi r^3$
$4\ x\ \pi\ x\ 50^2\ x\ 5 \ :\ \frac{3}{4}\ x\ \pi\ x\ 55^2$
$157080 \ :\ 392012$
$1 \ :\ 2.5$
$0.2 \ :\ 0.5$

Elemental analysis
$2000 : 22.3\ ppm$
$2000 : 28.46\ ppm\ (correction\ for\ magnetite\ Mw)$
$2000 : 28.99\ ppm\ (correction\ for\ maghemite\ Mw)$

% Magnetite $= \frac{Magnetite\ ppm}{Polymer + Magnetite\ ppm} x\ 100 = \frac{28.46}{2028.46}\ x\ 100 = 1.40\ \%$

% Maghemite $= \frac{Maghemite\ ppm}{Polymer + Maghemite\ ppm} x\ 100 = \frac{28.99}{2028.99}\ x\ 100 = 1.42\ \%$

Magnetic saturation (from SQUID measurement) $= 3.76\ x\ 10^{-5}\ emu\ g^{-1}$

If magnetite $= \frac{emu}{g} = 91$ therefore $g = \frac{3.76\ x\ 10^{-5}}{91} = 4.13\ x\ 10^{-7}$
% Magnetite material $= \frac{4.13\ x 10^{-4}}{0.200413}\ x\ 100 = 0.20\ \%$
If magnetite $= \frac{emu}{g} = 80$ therefore $g = \frac{3.76\ x\ 10^{-5}}{80} = 4.7\ x\ 10^{-7}$
% Magnetite material $= \frac{4.7\ x 10^{-4}}{0.20047}\ x\ 100 = 0.23\ \%$
